# Behavioral Alterations of Spatial Cognition and Role of the Apolipoprotein E-ε4 in Patients with MCI Due to Alzheimer’s Disease: Results from the BDSC-MCI Project

**DOI:** 10.3390/jcm13185447

**Published:** 2024-09-13

**Authors:** Davide Maria Cammisuli, Virginia Bellocchio, Alessandra Milesi, Edoardo Nicolò Aiello, Barbara Poletti, Federico Verde, Vincenzo Silani, Nicola Ticozzi, Gloria Marchesi, Valentina Granese, Benedetta Vignati, Valeria Isella, Stefano Zago, Teresa Difonzo, Simone Pomati, Giovanni Porta, Stefania Cattaldo, Alessandro Mauro, Gianluca Castelnuovo

**Affiliations:** 1 Department of Psychology, Catholic University, 20123 Milan, Italy; davide.cammisuli1@unicatt.it (D.M.C.); gloria.marchesi03@icatt.it (G.M.); 2 Catholic University, 20123 Milan, Italy; virginiabellocchio12@gmail.com (V.B.); valentina.granese@unicatt.it (V.G.); benedetta.vignati01@icatt.it (B.V.); 3 Clinic Neurobiology Laboratory, IRCCS Istituto Auxologico Italiano, “San Giuseppe” Hospital, 28824 Piancavallo, VB, Italy; a.milesi@auxologico.it (A.M.); s.cattaldo@auxologico.it (S.C.); 4 Department of Neurology and Laboratory of Neuroscience, IRCCS Istituto Auxologico Italiano, 20149 Milan, Italy; e.aiello@auxologico.it (E.N.A.); b.poletti@auxologico.it (B.P.); f.verde@auxologico.it (F.V.); vincenzo@silani.com (V.S.); n.ticozzi@auxologico.it (N.T.); 5 Department of Oncology and Hemato-Oncology, Università degli Studi di Milano, 20122 Milan, Italy; 6 Dino Ferrari Centre, Department of Pathophysiology and Transplantation, University of Milan, 20122 Milan, Italy; 7 Department of Neurology, School of Medicine, University of Milano-Bicocca, 20126 Milan, Italy; valeria.isella@unimib.it; 8 Milan Centre for Neurosciences, 20133 Milan, Italy; 9 Fondazione IRCCS Ca’ Granda, Ospedale Maggiore Policlinico, University of Milan, 20122 Milan, Italy; stefano.zago@unimi.it (S.Z.); teresa.difonzo@policlinico.mi.it (T.D.); 10 Neurology Unit, Luigi Sacco University Hospital, 20157 Milan, Italy; simone.pomati@asst-fbf-sacco.it; 11 Department of Medicine and Surgery, University of Insubria, 21100 Varese, Italy; giovanni.porta@uninsubria.it; 12 “Rita Levi Montalcini” Department of Neurosciences, University of Turin, 10126 Turin, Italy; alessandro.mauro@unito.it; 13 Neurology and Neurorehabilitation Unit, IRCCS Istituto Auxologico Italiano, “San Giuseppe” Hospital, 28824 Piancavallo, VB, Italy; 14 IRCCS Istituto Auxologico Italiano, Clinical Psychology Research Laboratory, 20149 Milan, Italy

**Keywords:** Alzheimer’s disease, MCI due to AD, subjective cognitive decline, spatial navigation, Apolipoprotein E-ε4

## Abstract

**Background**: Beyond memory deterioration, spatial disorientation may occur along the continuum of normal aging—dementia of Alzheimer’s type. The present study aims at detecting behavioral disorders of spatial cognition in prodromal Alzheimer’s disease (AD) and verifying the association between Apolipoprotein E-ε4 (ApoE-ε4) genotype and gait patterns during a real-world naturalistic task. **Methods**: A sample of 58 elderly participants, of which 20 patients with mild cognitive impairment with CFS biomarker evidence of AD, 23 individuals with subjective cognitive decline (SCD), and 15 healthy controls (HCs), was tested by a modified version of the Detour Navigation Test (DNT-mv). Generalized linear models were run to explore the association between group belonging and wrong turns (WTs)/moments of hesitation (MsH) as behavioral disorientation scores of the DNT-mv as well as the effect of ApoE-ε4 genotype on time and walking speed registered by a smartphone app providing GPS tracking of body movement around urban environments. **Results**: Patients with MCI due to AD reported more WTs than individuals with SCD and HCs. Further, the ApoE-ε4 genotype determined a lower capacity in spatial information processing, influencing gait during naturalistic spatial navigation tasks. **Conclusions**: Behavior alterations of spatial cognition can be detected ecologically in prodromal AD. The use of technological solutions supporting gait analysis may help in corroborating the experimental observation.

## 1. Introduction

Alzheimer’s disease (AD) is a neurodegenerative disorder marked by a gradual decline in cognitive abilities, prominently featuring episodic memory loss and spatial disorientation (SD). Deterioration of spatial navigation (SN) abilities often presents early in AD and has a significant impact on patients’ quality of life since these abilities allow individuals to navigate environments independently, remember landmarks, locate objects, and memorize their position in space [1]. SN abilities mainly pertain to egocentric and allocentric strategies [2]. The existence of spatial representations to proficiently navigate familiar and unfamiliar environments has been introduced by Tolman [3], and there is currently a recognized distinction between an egocentric and an allocentric type of frame [1]. While the egocentric frame includes spatial information about the location of the individual in the environment, defining spatial position by using the navigator’s body as a point of reference, allocentric frames involve information about the position of objects and their reciprocal spatial relationships, independently from the observer’s location [1,4]. The effects of these representations can be dissociated behaviorally and in terms of their neural basis, with the hippocampus providing allocentric environmental representations, the parietal lobe egocentric representations, and the retrosplenial cortex and parietal-occipital sulcus allowing both types of representations to interact [5,6]. In addition, hippocampal and striatal systems process different aspects of environmental layout—boundaries and local landmarks, respectively [5,6]. Given the importance of SN abilities in everyday life for elderly independence from caregivers, understanding neural mechanisms of SD in patients at higher risk of developing AD can be relevant to improve diagnosis and suggest preventive therapeutic strategies [7].

The pathogenesis of AD is characterized by an insidious onset. The hallmarks of the disease consist of the presence of extracellular senile plaques containing insoluble β-amyloid peptide (Aβ) and intracellular neurofibrillary tangles composed of phosphorylated tau protein (P-tau) within neuronal cytoplasm [8]. Apart from old age, the ε4 allele of the Apolipoprotein E (ApoE) gene stands out as the most relevant genetic risk factor for AD, offering an opening for assessing subclinical behavioral changes in very early disease stages and in mild cognitive impairment (MCI), which represents the symptomatic predementia phase [9].

Substantial evidence indicates that ApoE plays a crucial role in AD pathogenesis, with the overall effect of ApoE-ε4 being to hasten the onset of Aβ deposition into amyloid plaques [10]. Apolipoproteins are responsible for transporting lipids into the bloodstream and also bind to the hydrophobic amyloid-β (Aβ) peptide, believed to trigger toxic events leading to synaptic dysfunction and neurodegeneration in AD. The ApoE genotype is determined by three isoforms -ε2, -ε3, and -ε4 linked to the polymorphisms T334C (rs429358) and C472T (rs7412) by amplification of the target sequences and analysis of melting curves. Possible nucleotide combinations determine the six genotypes of interest (ε2/ε2, ε2/ε3, ε2/ε4, ε3/ε3, ε3/ε4, and ε4/ε4).

On one hand, the ApoE-ε4 allele is a well-established risk factor for AD: individuals carrying one ApoE-ε4 allele face three-to-four-fold increased risk, and those with two ApoE-ε4 alleles have more than a ten-fold increased risk of developing AD [11]. On the other hand, the ε2 allele seems to be associated with protection against the disease, although this assumption remains unclear to date [12], also considering that recent literature has shown that this allele does not provide its typically protective effect when present alongside ε4 in individuals with the relatively rare ε2/ε4 genotype, who appear comparable to the more prevalent ε3/ε4 group [13].

The ApoE-ε4 genotype thus plays a key role in the neuropathological process underlying AD, and in its early symptomatic stages, this process primarily affects regions involved in spatial navigation, particularly the hippocampus and entorhinal cortex, which host neuronal groups responsible for locating objects in space and navigating through it [14]. In turn, the motor component of spatial cognition is represented by gait, which is why an impairment in spatial cognition can manifest as a loss of gait fluidity and motor slowing.

Slow gait itself has also been linked to a heightened risk of cognitive impairment and is considered a robust predictor of dementia in both population-based and clinical investigations [15,16]. It has been suggested that the combination of ApoE-ε4 genotype and slow gait represents a greater risk factor for cognitive impairment [17]. Moreover, it has been previously shown that carrying at least one copy of the ApoE-ε4 gene represents a risk factor for gait impairment in MCI [18]. However, limited research to date has been conducted using modern technologies able to precisely assess walking patterns in MCI and AD patients [19] during ecological tasks, and spatial navigation-related abilities have been often investigated without technological device support [20]. Further, previous studies have solely focused on AD patients [21], without considering its preclinical phase, i.e., the subjective cognitive decline (SCD) [22,23,24]. Self-reported SN complaints also have been reported in individuals with SCD, too [25]. However, investigations examining the association between ApoE-ε4 carrier status and SCD have produced mixed results [26].

### Aims of the Study

The present study aimed (i) to assess whether MCI due to AD presents with behavioral disorders of spatial cognition greater than individuals with SCD and healthy controls, and (ii) to verify if the ApoE-ε4 can predict significant changes on gait parameters measured by a GPS mobile application recording body movement during a naturalistic SN task.

## 2. Materials and Methods

### 2.1. Participants

The study included 20 patients with MCI due to AD, 23 individuals with SCD, and 15 healthy controls. Patients with MCI due to AD and individuals with SCD were diagnosed according to the criteria outlined by Petersen et al. [9], Dubois et al. [27], and Jessen et al. [22], respectively. Additionally, healthy controls (HCs) were recruited via advertisements disseminated in social centers for senior citizens of the Milan community (Lombardy Region, Italy) as well as through participation in conferences and webinars related to AD research and prevention.

All participants met specific inclusion criteria, including an age range of 60 to 85 years, a minimum education level of 5 years, the absence of dementia, and proficiency in basic information and communication technology (ICT) skills. Exclusion criteria encompassed a history of alcohol or substance abuse, diagnosis of neurological or psychiatric disorders, or any other medical conditions that could potentially affect cognitive function, such as head injury, vision impairment, or motor disabilities.

The study protocol has been registered in ClinicalTrials.gov (ID: NCT05944601). The study was first approved by the Auxologico Ethics Committee (2023_05_30_BDSC-MCI_IAI). Then, an amendment to the original protocol was also approved by the Ethics Committee Lombardy 5 on session 20 February 2024.

All participants signed the informed consent to participate, and the research was conducted in accordance with the 1964 Helsinki Declaration and standards for good research practice. All participants were covered by the AON insurance policy (P4427 Trial Access BDSC-MCI) for potential person damage (e.g., falling, injury, etc.) during the outdoor experimental task.

### 2.2. Measurements

#### 2.2.1. Clinical Assessment

Before administering the ecological task, we assessed participants at the Mosé Bianchi Hospital, IRCCS, Istituto Auxologico Italiano, adjacent to the Pontificio Istituto Missioni Estere (PIME) urban garden (81, Monte Rosa Street, 20149 Milan, Italy), in which participants underwent the naturalistic SN task (See below; for a complete description of the task, please see Cammisuli et al. [14]). A global cognition screening was administered using either the Mini Mental State Examination (MMSE) [28,29] or the Montreal Cognitive Assessment (MoCA) [30], given the fact that our study had a multi-center recruitment. For participants assessed by the MMSE, we adopted Aiello and colleagues’ [31] conversion norms to obtain MoCA scores. Affective status was also evaluated by the Geriatric Depression Scale (GDS) [32].

#### 2.2.2. ApoE Genotyping

Genomic DNA was extracted from buccal brushes using the Gentra Puregene Buccal Cell Kit (Qiagen, Valencia, CA, USA). Sample processing was performed according to the manufacturer’s instructions, using the ApoE Real Time (FRET) kit (Nuclear Laser Medicine, Settala, MI, Italy).

According to the genetic risk, we then divided the whole sample into high-risk (h-R) and low-risk (l-R) subgroups by dividing those participants carrying at least one copy of the ApoE-ε4 (i.e., ε2/ε4, ε3/ε4, ε4/ε4) from non-carriers (ε2/ε2, ε2/ε3, ε3/ε3).

#### 2.2.3. Gait Assessment

Before the navigation of the PIME urban garden, all participants were equipped with the Howdy Senior System© (Comftech S.r.l., Monza, Lombardy, Italy) at the hospital, a sensory garment with a connected smartphone app for continuous monitoring of physiological data. The system has been recently implemented by a GPS mobile application for gait analysis investigation, determining GPX tracks of body position and movements around urban environments. Such tracks were elaborated by another smartphone app, the ‘Aptive’ one. Beyond ‘Latitude’, ‘Longitude’, ‘Altitude’, and ‘Direction’ corresponding to gait orientation towards cardinal points, the app was able to record ‘Time’, as the number of minutes to complete a predefined route (minutes, min), and ‘Speed’, as walking velocity provided by geolocation (meters per second, m/s). Prior to starting the naturalistic SN task, the smartphone equipped with the app was given directly to the participants, who kept it with them for the entire duration of the test. This ensured the accuracy of gait measurements recorded by the Aptive app.

Participants were required to complete the Detour Navigation Test-modified version (DNT-mv). Unlike the original paradigm described by Puthusseryppady and colleagues [20], this ecological task is designed to assess SN alterations in patients with prodromal AD as they navigate an unfamiliar location. At this stage, patients are in fact still familiar with their neighborhoods and do not become disoriented during routine community walks, unlike those with advanced dementia.

After donning the sensory garment at the hospital, participants were accompanied to the adjacent urban park (for a visual depiction, please see Cammisuli et al. [14]). This park, encircled by buildings and away from urban roads, was chosen to ensure participant safety and minimize confounding variables. Participants were first guided along a path from a start point to a destination (i.e., Route A outward) while being instructed to observe landmarks along the way. Mid-route, they were asked to perform a simple arithmetic task (subtracting 7 from 100) without stopping, simulating urban distractions (e.g., car horns, background noise, a familiar person greeting them, or a phone call). Upon reaching the destination, participants were shown 12 pictures and asked to identify whether they were the landmarks encountered (n.6) or distractors (n.6) similar to the landmarks.

Next, participants had to navigate back to the starting point on their own (i.e., Route A return). If they deviated from the original path, the investigator provided feedback to help them correct their direction. This task primarily involves egocentric sequential navigation, where participants rely on self-centered information and visual processing of a sequence of turns in relation to their body movements. Subsequently, participants were asked to reach the destination point again (i.e., Route B outward). However, at the first intersection, they were unexpectedly required to find an alternative route back (i.e., Route B return) that did not overlap with Route A. This task demands an allocentric strategy, relying on a cognitive map of the urban garden. For the Route B return, the investigator followed without offering feedback. The entire DNT-mv took approximately 20 min to administer.

As disorientation scores of the DNT-mv, wrong turns (WTs) and moments of hesitation (MsH) were calculated both for Route A return and Route B return and summed up together. Wrong turns and MsH represent behavior alterations of spatial cognition, indicating the difficulty in recalling sequential body turns in a route-retracing task and the inability to recognize one’s whereabouts (i.e., forming a cognitive map of the explored environment), respectively. Time and speed variables were registered by the Aptive app (i.e., Time Route A return, TRA; Time Route B return, TRB; Speed Route A return, SRA; Speed Route B return, SRB) and separately calculated for each route (A return and B return).

### 2.3. Statistics

All outcome measures were checked for normality by computing skewness and kurtosis values (judged as indexing non-normal distributions if > |1| and |3|, respectively [33]) and by visually inspecting histograms and quantile-quantile plots. All measures proved to be heavily right-skewed and overdispersed; hence, generalized linear models assuming an underlying Negative Binomial or Gamma distribution—for integer and non-integer data, respectively—were run when addressing them as outcomes [34]. Within both Negative Binomial and Gamma models, a logarithmic link function was employed. Within all the abovementioned models, Group and Genetic risk were entered as main terms, while age, education, and sex were covered.

Analyses were run via Jamovi 2.3 (The jamovi project, 2023). Within post-hoc comparisons, significant thresholds were Bonferroni-corrected.

## 3. Results

Participants’ demographic and cognitive measures are reported in Table 1.

The three groups were balanced for age (*F*(2, 55) = 2.46, *p* = 0.095), gender (χ^2^(2) = 1.89; *p* = 0.389), education (*F*(2, 55) = 0.78, *p* = 0.462), and depressive symptoms (GDS; *F*(2, 51) = 2.88, *p* = 0.065), whereas significant differences were detected in terms of genetic profiles (χ^2^(8) = 17.60; *p* = 0.024) and thus level of genetic risk (χ^2^(2) = 13.35; *p* = 0.001)—with a trend towards a higher proportion of at least one ε4 allele in MCI patients and ε3/ε3 genotypes being the most represented in HCs and SCD individuals.

A significant discrepancy was also detected as to MoCA scores (*F*(2, 55) = 19.89, *p* < 0.001), with MCI patients performing worse than both SCD (*p* < 0.001) and HC individuals (*p* = 0.007) and SCD patients performing better than HCs (*p* = 0.038), as expected.

As for behavioral disorientation scores, significant between-group differences were detected, with MCI patients reporting more WTs than both HC (*p* < 0.001) and SCD individuals (*p* = 0.005) and significantly more MsH than HCs (*p* = 0.009) (Table 2). Significant between-group differences were also found as for TRA and were accounted for, at post-hoc comparisons, by MCI patients showing worse performance than HCs (*p* < 0.001) and SCD individuals (*p* = 0.003), and by the SCD group performing significantly worse than HCs (*p* = 0.035). In terms of SRA, HCs performed better than both MCI (*p* = 0.002) and SCD patients (*p* = 0.005). Post-hoc comparisons addressing TRB and SRB revealed that MCI patients took more time to complete the path than HCs (*p* = 0.006), while HCs walked significantly faster than both SCD (*p* < 0.001) and MCI patients (*p* < 0.001).

**Figure 1:**
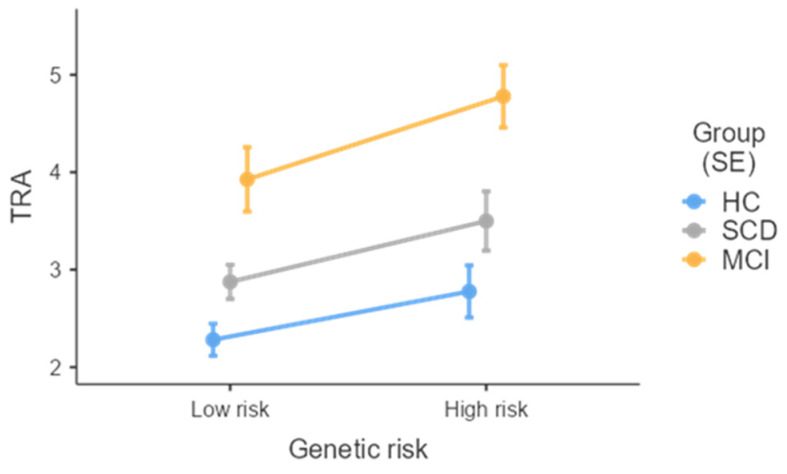
Effect of Genetic risk on TRA split by group. TRA = Time Route A return; HC = healthy controls; SCD = subjective cognitive decline; MCI = mild cognitive impairment; SE = standard error.

As far as the genetic component is concerned, a significant effect of genetic risk was found regarding TRA, with the h-R group reporting a longer time to complete the path (*M* = 3.59; *SE* = 0.23) when compared to the l-R group (*M* = 2.95; *SE* = 0.14) (Figure 1).

**Figure 2:**
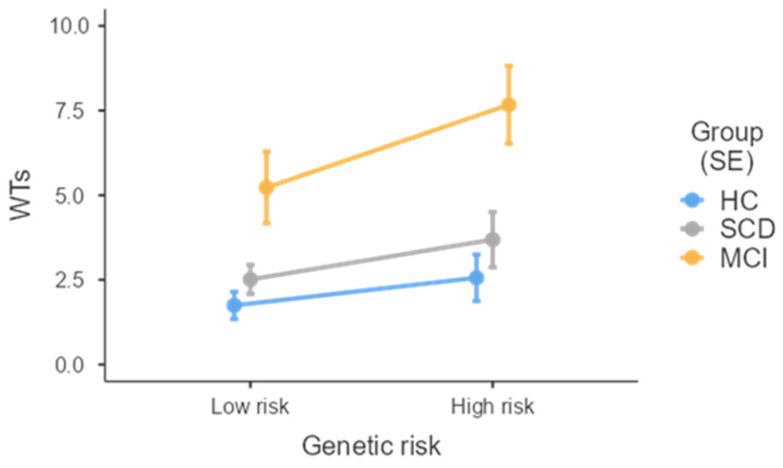
Effect of Genetic risk on WTs split by group. WTs = wrong turns; HC = healthy controls; SCD = subjective cognitive decline; MCI = mild cognitive impairment; SE = standard error.

A marginally significant effect of genetic risk was also detected concerning WTs, with h-R subjects reporting overall more WTs (*M* = 4.17; *SE* = 0.66) when compared to l-R ones (*M* = 2.84; *SE* = 0.38), net of group belonging (Figure 2).

**Figure 3:**
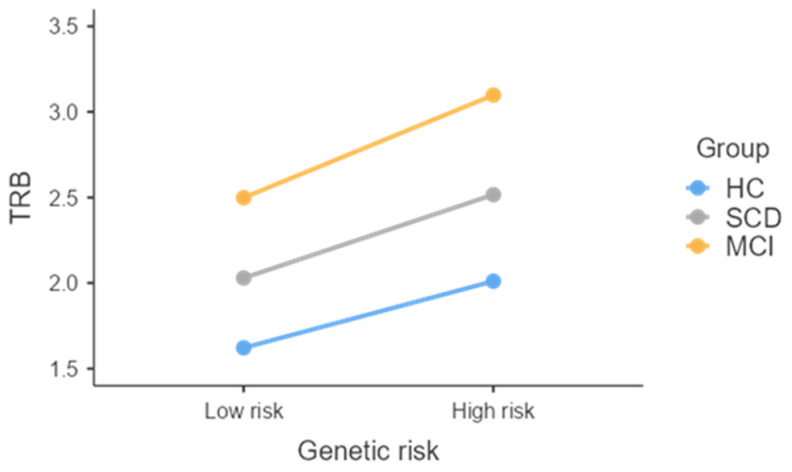
Effect of Genetic risk on TRB split by group. TRB = Time Route B return; HC = healthy controls; SCD = subjective cognitive decline; MCI = mild cognitive impairment; SE = standard error.

Similarly, another marginally significant main effect was detected on TRB, carried by differences in the time needed to travel the route between the h-R group (*M* = 2.50; *SE* = 0.22) and the l-R one (*M* = 2.02; *SE* = 0.13) (Figure 3).

## 4. Discussion

Early detection of AD has gained significant importance as it enables earlier intervention and potentially more effective treatment. Results confirm our previous investigation [35], which had shown that MCI due to AD exhibits worse SN performances in a real-world navigation task than HCs. However, the present study represents a step further, demonstrating that MCI due to AD also reported more difficulty (WTs) in recalling sequential body turns in a route-retracing task than SCD, pointing out a visuospatial long-term memory impairment. SCD fits well into the trajectory towards AD, thus representing an intermediate phase between normal aging and MCI [36]. Remarkably, individuals with SCD also completed the route-retracing task by consuming more time (TRA) than HCs but less time than MCI. Such a trend highlights that cognitive deterioration due to progressive neuropathological damage due to AD also reflects a gradual decline in visuospatial processing speed during navigation.

SCD is a heterogeneous entity, and its heterogeneity has also been investigated from the perspective of SN performance. Chen and colleagues [37] demonstrated that part of the neural structural basis underlying SN, i.e., the basal forebrain and the right hippocampus, showed lower volumes in SCD individuals, with poor SN performances compared with SCD individuals with preserved SN abilities. These individuals mainly showed atrophy in the so-called nucleus basalis of Meynert, a key structure for cholinergic input to the medial prefrontal, cingulate, retrosplenial, and visual cortices [37]. These areas, particularly the medial prefrontal cortex, play a crucial role in many aspects of SN, including route planning, route plan updating, and path selection [37,38]. Moreover, the posterior cingulate and retrosplenial cortex have been associated with integrating hippocampal-related allocentric spatial information and parietal-related egocentric spatial information, also facilitating the flexible transition between these two navigation strategies [1]. Both the basal forebrain and hippocampus are areas early affected by AD pathology. In line with this, SCD individuals who exhibited SD also report a significantly higher likelihood of progression to MCI compared with those who correctly performed in SN paradigms [37].

Regarding the genetic risk represented by carrying the ApoE-ε4 allele, we found that the h-R group negatively influenced the visuospatial processing speed during navigation (TRA) regardless of group belonging. This result is in accordance with previous research showing that ApoE-ε4 carriers perform significantly poorer than ApoE-ε4 non-carriers both on computer-based SN tasks [39] and in a real-space navigation setting [40], suggesting that the presence of ApoE-ε4 significantly influences egocentric-based navigation based on sequential body turns learning. It has also been reported that patients with amnestic MCI (aMCI) carrying the combination of both ApoE-ε4 and the brain-derived neurotrophic factor (BDNF) Val66Met polymorphism showed a significantly more pronounced egocentric spatial navigation impairment compared to those not carrying these two gene polymorphisms [41].

To identify the visuospatial processing speed deficit during navigation in h-R individuals, the use of technological devices was crucial for accurately recording gait measurements within a limited time frame in an easy and comfortable manner. This makes our report an original scientific work, given the paucity of studies present in the literature to date. Our investigation also contributes to enriching the epidemiological data, considering that population allele frequencies of the ApoE vary by geographic region all over the world, among healthy subjects and AD patients [42]. Studies on European countries commonly found a distinct decrease in ε4 allele frequencies from North to South, along with an opposite trend for ε3 frequencies [43,44]. Recent research regarding the prevalence of ApoE-ε4 specifically in the Italian general population is limited. Corbo and colleagues [44] reported similar frequencies for the ε2, ε3, and ε4 alleles in Central and Southern Italy, with significantly lower ε4 frequencies found in the Sardinia region. In our sample, we reported a significantly high prevalence of at least one ε4 allele in MCI patients. These data are not entirely consistent with those already present in literature, where the lack of a strong association between ApoE-ε4 and AD has been previously reported [45,46], with some exceptions [47,48].

However, our study had some limitations. The three groups (MCI, SCD, and HCs) were not perfectly balanced, even if they were well-matched in terms of age, education, gender, and depressive symptoms. We also need to point out that, while we assessed the affective status of our participants, we did not evaluate their anxiety levels, which could be useful to address in order to further eliminate potential differences between groups. Furthermore, by using the Italian norms from Aiello and colleagues [31], we had to convert the MMSE scores into MoCA scores for those participants that were not assessed by the latter instrument, since our study was a multi-center investigation recruiting participants from three different sites. Additionally, the dichotomous stratification of genetic risk (i.e., h-R vs. l-R) due to the limited sample did not allow us to conduct more detailed analyses, for example, to verify the potential protective effect of ApoE-ε2 on visuospatial processing speed. Relatedly, the readership has to bear in mind that the restricted sample size might have biased the results of our inferential analyses, which should be thus addressed as preliminary. Future investigations addressing a larger sample of participants and aiming at replicating/disconfirming the current results are thus warranted. Lastly, although ApoE-ε4 represents the strongest genetic risk factor for AD [49], as genetic research goes on, studies have found suggestive links between late-onset AD and a number of other genes, such as ABCA7, CLU, CR1, PICALM, PDL3, TREMZ, and SORL1 [50], that might influence spatial navigation performances.

## Figures and Tables

**Table 1:** Participants’ sociodemographic, clinical, neuropsychological, and behavioral measures.

	HC	SCD	MCI	P
N	15	23	20	-
Sex (male/female)	7/8	9/14	12/8	n.s ^a^
Age (years)	74.3 ± 7.7 (64–85)	70.2 ± 7.4 (56–80)	74.0 ± 4.8 (62–82)	n.s ^b^
Education (years)	12.2 ± 2.7 (8–18)	12.0 ± 4.0 (8–18)	10.7 ± 4.6 (5–18)	n.s ^b^
ApoE genotype				0.024 ^a^
ε2/ε3 ε2/ε4 ε3/ε3 ε3/ε4 ε4/ε4	0 1 12 1 1	1 1 17 4 0	1 1 5 11 2	- - - - -
Genetic risk (low/high)	12/3	18/5	6/14	<0.001 ^a^
GDS	5.0 ± 4.2 (0–13)	9.3 ± 5.4 (1–21)	9.6 ± 7.5 (0–21)	n.s. ^b^
MoCA (adjusted scores) ^c^	24.7 ± 2.3 (21.6–29.7)	27.8 ± 3.0 (18.6–30.0)	20.7 ± 5.0 (10.8–27.6)	SCD > HCs > MCI ^b^
WTs	1.9 ± 2.1 (0–7)	2.8 ± 2.5 (0–10)	7.4 ± 4.2 (2–15)	MCI > HCs & SCD ^d^
MsH	13.0 ± 21.2 (0–78)	26.6 ± 45.1 (0–170)	105.7 ± 84.7 (0–342)	MCI > HCs ^d^
TRA	2.4 ± 0.6 (1.6–4.0)	3.1 ± 0.9 (2.0–5.5)	4.5 ± 1.6 (2.2–8.0)	MCI > SCD > HCs ^d^
SRA	0.6 ± 0.2 (0.3–0.9)	0.5 ± 0.1 (0.3–0.8)	0.4 ± 0.2 (0.2–0.8)	HC > MCI & SCD ^d^
TRB	1.7 ± 0.38 (1.3–2.6)	2.1 ± 0.8 (1.3–4.3)	3.1 ± 1.7 (1.4–7.3)	MCI > HCs ^d^
SRB	0.7 ± 0.2 (0.3–1.0)	0.5 ± 0.1 (0.3–0.6)	0.5 ± 0.2 (0.2–0.9)	HC > MCI & SCD ^d^

GDS = Geriatric Depression Scale; MoCA = Montreal Cognitive Assessment; WTs = wrong turns; MsH = moments of hesitations; TRA = Time Route A return; SRA = Speed Route A return; TRB = Time Route B return; SRB = Speed Route B return; ^a^ χ^2^-statistic for independent samples; ^b^ Tukey-adjusted *p*-values for an *F*-statistic; ^c^ Santangelo et al.’s [30] correction grid. ^d^ Dwass-Steel-Critchlow-Fligner-adjusted *p*-values for a Kruskal-Wallis χ^2^-statistic.

**Table 2:** Results of the models testing the effect of Genetic risk and Group on gait analysis measures.

		χ^2^	*p*
WTs			
	Genetic risk	3.22	0.073
	Group	17.97	<0.001
MsH			
	Genetic risk	2.04	0.153
	Group	8.76	0.013
TRA			
	Genetic risk	5.57	0.018
	Group	28.40	<0.001
SRA			
	Genetic risk	0.23	0.635
	Group	14.93	<0.001
TRB			
	Genetic risk	3.35	0.067
	Group	8.80	0.012
SRB			
	Genetic risk	0.01	0.926
	Group	24.56	<0.001

WT = wrong turns; MsH = moments of hesitation; TRA = Time Route A return; SRA = Speed Route A return; TRB = Time Route B return; SRB = Speed Route B return.

## Data Availability

Requests to access the datasets should be directed to correspondence author (gianluca.castelnuovo@auxologico.it), due to access restrictions.
